# Radiotherapy tattoos: Women's skin as a carrier of personal memory—What do we cause by tattooing our patients?

**DOI:** 10.1111/tbj.13591

**Published:** 2019-09-16

**Authors:** Torsten Moser, Menna Creed, Robyn Walker, Gernot Meier

**Affiliations:** ^1^ Vision RT London UK; ^2^ EKIBA Karlsruhe Germany

For decades, tattoos and temporary skin marks have been used in the delivery of radiation therapy.^1^, ^2^ Women suffer not only by the knowledge of the disease, but they are also marked with reminders of the experience by an increasingly outdated positioning technique (Cravo Sá, Fermento et al 2018). On one hand, tattoos help radiation therapists to effectively and accurately position patients. On the other hand, the especially gendered connotation of breast cancer^3^ means that women may experience the illness as an attack on their femininity^4^ with tattoos and temporary marks providing a visible and constant reminder of this disease and the patient's treatment and fight against it. Consequently, they can be a violation of their most prominent physical attribute of womanhood.

This leads to the question: How do the patients themselves feel about their marks? Would they avoid skin marks if they had the chance? Over the past several years, the technique of SGRT—Surface Guided Radiation Therapy—has been shown to offer a substitute for skin marks, delivering at least equivalent accuracy in patient positioning,^5^, ^6^, ^7^ and is increasingly used in clinical practice for this and other reasons. Between February and August 2018, data were collected from members of the Young Survival Coalition (YSC). YSC was founded in 1998 and is the premier organization dedicated to the critical issues unique to women who are diagnosed with breast cancer under the age of 40. Affected women were interviewed to understand their needs and feelings.

Women attending the 2018 annual YSC meeting in Orlando, Florida were selected randomly and asked to complete a questionnaire. This was either performed immediately using a tablet computer or after the meeting online. A total of 142 women answered the survey (Table 1). Four women did not receive radiotherapy during their treatment and were therefore excluded from further analysis. Depending on the facilities procedures, tattoos and/or marks were placed on every surveyed patient according to the relative treatment region.

**Table 1:** Age distribution of the patients

Age (y)	18‐24	25‐34	35‐44	45‐54	55‐64	65‐74	>75
No.	1	34	63	39	3	2	0

To reduce bias, open questions were asked, with linguistic analysis^8^ used retrospectively for classification of answers. Responses such as “annoying, felt awful, concerned” were the most frequent negatives, with words like “fine, it's OK, and helping” most frequent positives. The data were then examined for trends.

**Figure 1:**
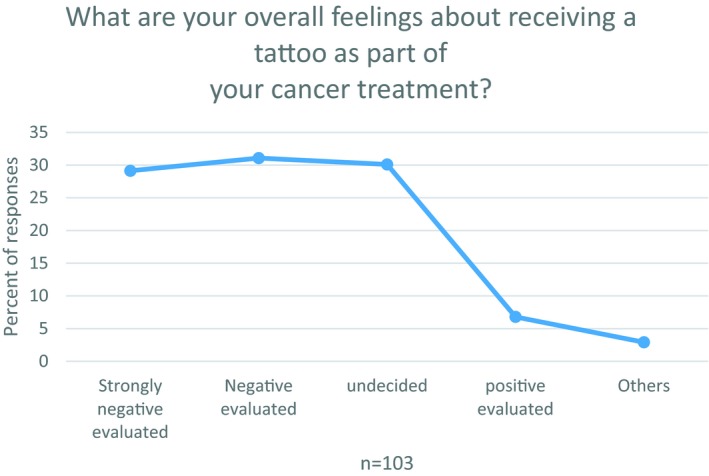
Overall feeling tattoos

Data from the surveys demonstrated a very clear trend for the central question “What are your overall feelings about receiving a tattoo as part of your cancer treatment?” While only a few women have positive feelings with them, and some are undecided, the majority scored “negative” or “very negative” (Figure 1). If one assumes that the online survey has interviewed women from many different life and work contexts in the US, the result suggests universal negative sentiment toward treatment‐related tattoos: Today, tattoos are a part of contemporary culture and sometimes a means of remembrance.^9^ However, remembrance here was typically not the positivity of the possible recovery but the memory of the disease. The skin had become a carrier of personal memory.

**Figure 2:**
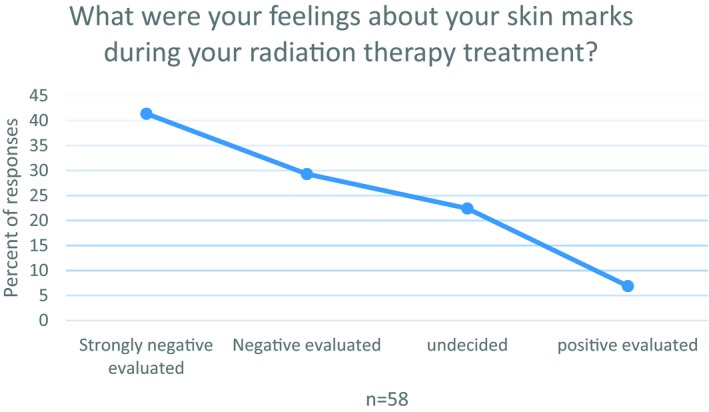
Overall feeling temporary marks

The relating question for temporary marks reveals similar trends (Figure 2). Only 7% describe positive feelings. While 22% are undecided, approx. 70% of the women score them as negative.

Reinforcing these findings, 78% of the interviewed women stated they would choose treatment which avoided tattoos and/or marks, even if additional efforts were required, for example, additional costs, distance, time for travel etc (Table 2). Respondents were asked if SGRT technology had been available during their cancer treatment, how much they would have paid to avoid tattoos and/or marks by traveling to a treatment center using SGRT technology:

**Table 2:** Additional efforts to avoid marks/tattoos

One‐way trip	Mean ± 1 std
Additional distance in miles	45.5 ± 26.4
Additional travel time in minutes	39.3 ± 22.2
Additional cost of journey (in US $)	37.5 ± 25.3

There is no data base collecting this kind of information for a global analysis, and data in this paper are based on a survey around one YSC meeting. Therefore, the number of participants is limited, and the cross section might not be representative for all states or countries. Nevertheless, a clear trend can be observed with patients strongly desiring avoidance of permanent or temporary skin marking. For years, patients have been positioned by aligning their marks to the room lasers. Recent publication shows equal or even better accuracy in daily alignment for patients being positioned using SGRT technology as compared to conventional methods using marks and lasers referring to kV CBCT as ground truth.^10^ Therefore, SGRT technology can be considered as a desirable replacement for tattoos/marks: positioning patients with a high level of patient safety, improved accuracy, and without interfering with women's dignity. Further research must address the question of which narratives are transported here and which functions do these narratives have?

Women treated with radiotherapy for breast cancer were interviewed to find trends in their feelings about the procedure to mark their body for positioning purposes. Around 70% had negative feelings about this involuntary body modification. This result is especially astonishing as nowadays, many people consider tattoos as part of contemporary culture. The same mechanism of intentional display regarding voluntary skin marks results in a negative, visible memory of disease and women are willing to dedicate significant resources to avoiding it. Tattooless and body markerless positioning technique would therefore help women to overcome this additional burden.
